# Impact of Sandblasting and Plasma Electrolytic Oxidation on Surface Quality of Dental Implants

**DOI:** 10.17691/stm2023.15.6.05

**Published:** 2023-12-27

**Authors:** L.I. Zaynullina, R.G. Farrakhov, I.A. Ramazanov, R.Z. Khamatdinov, V.S. Dyuryagin, E.V. Parfenov

**Affiliations:** PhD, Associate Professor, Department of Materials Science and Physics of Metals; Ufa University of Science and Technology, 12 Karl Marx St., Ufa, Republic of Bashkortostan, 450008, Russia; PhD, Associate Professor, Department of Electronic Engineering; Ufa University of Science and Technology, 12 Karl Marx St., Ufa, Republic of Bashkortostan, 450008, Russia; Engineer, Nanotech Center; Ufa University of Science and Technology, 12 Karl Marx St., Ufa, Republic of Bashkortostan, 450008, Russia; Assistant, Department of Materials Science and Physics of Metals; Ufa University of Science and Technology, 12 Karl Marx St., Ufa, Republic of Bashkortostan, 450008, Russia; Director; NS Technology LLC, 26B Entuziastov St., Chelyabinsk, 454080, Russia; DSc, Head of the Department of Materials Science and Physics of Metals; Ufa University of Science and Technology, 12 Karl Marx St., Ufa, Republic of Bashkortostan, 450008, Russia

**Keywords:** plasma electrolytic oxidation, Ti alloys, sandblasting, biocompatible surface, corundum, dental implants

## Abstract

Titanium alloys have high biocompatibility, and, therefore, they are widely used in the production of implantable medical devices. Implants, in turn, must have certain surface properties for a positive osseointegration. To improve biocompatibility, as well as cell viability, numerous implant surface modifications have been proposed in order to improve topography, roughness parameters, and surface layer chemical and phase compositions.

The most common type of surface treatment for dental implants involves sandblasting with aluminum oxide Al_2_O_3_ (corundum). However, aluminum is not a biocompatible element, and it can contribute to development of various diseases. Currently, the method of plasma electrolytic oxidation is being actively developed to ensure formation of a biocompatible TiO_2_-based oxide coating on the surface of titanium implants.

**The aim of the study** was to establish the residual aluminum content in the surface layer of dental implants after sandblasting and subsequent plasma electrolytic oxidation to justify the effective process sequence in serial production of dental implants.

**Materials and Methods:**

The research was conducted to establish the residual content of aluminum in the surface layer of the NCTi implant subjected to two surface treatment methods: sandblasting and plasma electrolytic oxidation following the sandblasting.

**Results:**

Sandblasting with Al_2_O_3_ particles leads to fixation of such particles with Al weight fraction of 2.67±0.79% in the surface layer of the implant. Treatment of a dental implant using plasma electrolytic oxidation helps to reduce the Al weight fraction in the surface layer to 0.33±0.08% and significantly improves the implant corrosion resistance with a decrease in corrosion currents by an order of magnitude.

## Introduction

The characteristics of the implant surface significantly influences the osseointegration process [1]. Implantable medical devices are often made of titanium-based alloys [2]. Cell reactions directly depend on the chemical and physical characteristics of the implant, in particular, on the particle size, chemical composition, and surface morphology, as well as on the geometry of internal and external threads [3]. Human mesenchymal stem cells have poor adhesion to a smooth titanium surface; this can lead to the formation of a fibrous tissue layer between the implant and the surrounding bone, and, thus, to the process of fibro-osseous integration with subsequent development of mucositis and peri-implantitis with a loss of the dental implants [4, 5]. Multiple modifications to the implant surface were proposed to solve this problem and to increase the biocompatibility, as well as the cell viability; the said modifications affect the topography, roughness characteristics, and surface layer chemical composition [6, 7].

The known surface treatment methods that improve osseointegration include sandblasting and subsequent acid-enhanced chemical etching, which are currently considered to be the most effective methods [8]. Sandblasting is the directed impact of an abrasive material at high-pressure blast on the surface of the implant to create a surface with a specified roughness (R_a_ range from 1 to 3 μm) [9]. Sandblasting the surface with aluminum oxide (corundum) is the most wide-spread process. However, this treatment leaves alumina residue on the surface of the implant, which should be treated with great caution, as the residue can lead to the surrounding tissue destruction. Hence, one recommends procedures that eliminate blasting with aluminum oxide or reduce its effect on the composition of the surface layer [10].

Along with sandblasting, plasma electrolytic oxidation (PEO) of the implant surface is a topical method to create an oxide coating with improved biocompatibility [11]. With this method applied, the value of surface roughness R_a_ is maintained at the level of 1.0–1.5 μm, and the coating with a thickness of 10–20 μm has a porous structure and provides good corrosion resistance [12]. According to recent studies, PEO-coated dental implants are characterized by improved biocompatibility [13].

**The aim of the study** was to establish the residual aluminum content in the surface layer of dental implant subjected to two surface treatment methods: sandblasting, and plasma electrolytic oxidation following the sandblasting, to justify the effective process sequence in serial production of dental implants.

## Materials and Methods

NCTi implants (n=6), which were manufactured by NS Technology LLC (Chelyabinsk, Russia) from Grade 4 titanium, were selected as samples for the study. The dimensions of the implants were ø4×10 mm. The surface treatment was conducted by two different methods: sandblasting and plasma electrolytic oxidation after the sandblasting.

The sandblasting of the implants was performed in an injector-type abrasive blasting unit at NS Technology LLC by blowing with aluminum oxide Al_2_O_3_ (corundum) particles with a grain size of M32 according to the GOST 3647-80 standard under a compressed air pressure of 3–4 kgf/cm^2^ and a flow rate of 0.5–0.7 m^3^ per minute.

After the sandblasting, the implant samples were cleaned in an ultrasonic bath in isopropyl alcohol for 5 min. Then, three samples were subjected to PEO, after which they were washed in distilled water in an ultrasonic bath for 5 min.

Experiments with PEO were conducted at the Ufa University of Science and Technology (Russia) using an automated technological unit with a power of 50 kW, which allowed software control of the PEO parameters and keeping them at a specified level with high accuracy. The data acquisition system of the automated unit is based on the L-Card L-502 board (L-Card, Russia), and automated process control software is based on the LabVIEW academic version [14].

Plasma electrolytic oxidation was conducted in a 5-L plastic container, with a stainless-steel heat exchanger, also serving as a cathode. Here, the processed implant sample acted as an anode. Microcontroller system maintained the electrolyte temperature at 20±1°C. The PEO was performed with a pulsed bipolar mode, subject to stabilization of the pulse voltage [12], for 2 min. The treatment was carried out in an alkaline phosphate electrolyte with a conductivity of 15.3 mS/cm.

The developed coatings on the implant samples were examined with magnifications of ×20, ×500, ×1000 on a JSM-6490LV scanning electron microscope (SEM; JEOL, Japan) having an attachment for elemental microanalysis INCAX-Sight (Oxford Instruments, UK). At least 8 measurements of the elemental content mapping were made on an image with dimensions of 1200×1200 and 400×400 μm.

Electrochemical characteristics were studied using R-5X potentiostat (Elins LLC, Russia) in the Ringer’s solution (pH 7.4) in a 100-ml three-electrode cell with a silver chloride reference electrode (E_0_=0.222 V) and a graphite counter electrode. Polarization curves were measured in the range from –350 to +1000 mV with respect to the open circuit potential with a scanning speed of 0.25 mV/s. The free corrosion potential and corrosion current were calculated using the Tafel section of the cathodic branch of the polarization curve. Polarization resistance was determined by the slope of the polarization curve within ±10 mV with respect to the free corrosion potential. All tests were performed three times per sample type to determine the standard deviation.

### Statistical data processing

Images of the implant surface were analyzed by SEM with elemental microanalysis (at least 8 measurements from various places on the surface). The average value of the parameters and standard deviation were determined using the Microsoft Excel 2017 software package.

## Results

**Figure 1.**
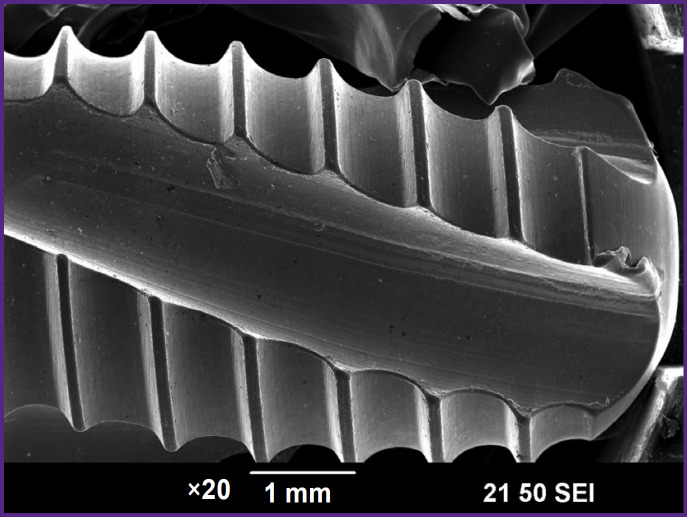
Appearance of the NCTi implant after mechanical treatment

The appearance of the NCTi implant after mechanical treatment is shown in Figure 1. Such processing results in burrs formation due to plastic deformation of the material during cutting.

**Figure 2.**
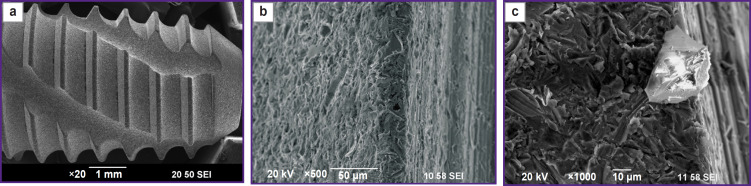
SEM image of the NCTi implant after sandblasting: (a) appearance; (b) image of the implant surface; (c) corundum particle embedded in the surface layer after treatment

To remove burrs and create a rough surface, which is better for bone cells to attach during osseointegration (Figure 2 (a)), sandblasting was used. The rough surface is formed as a result of mechanical impact of the sharp edges of corundum particles (Figure 2 (b)). In an SEM image with the magnification of ×1000, one can see corundum particles stuck in the surface layer (Figure 2 (c)).

**Figure 3.**
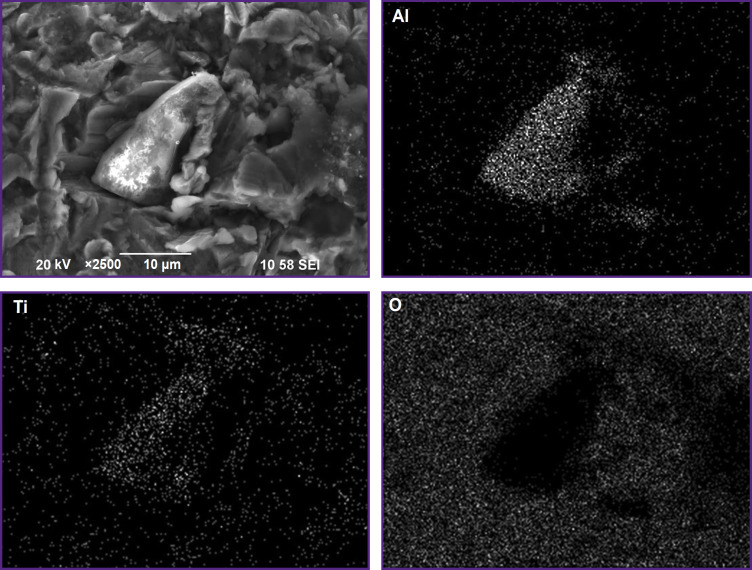
Mapping of Al_2_O_3_ corundum particles after sandblasting of a titanium implant, ×2500

An example of a particle identification as a result of the elemental mapping is shown in Figure 3. Analysis of the elemental composition confirms that the particle is an Al_2_O_3_ crystal. The size of corundum particles ranges from 10 to 30 μm which is consistent to the M32 fraction grade.

**Figure 4.**
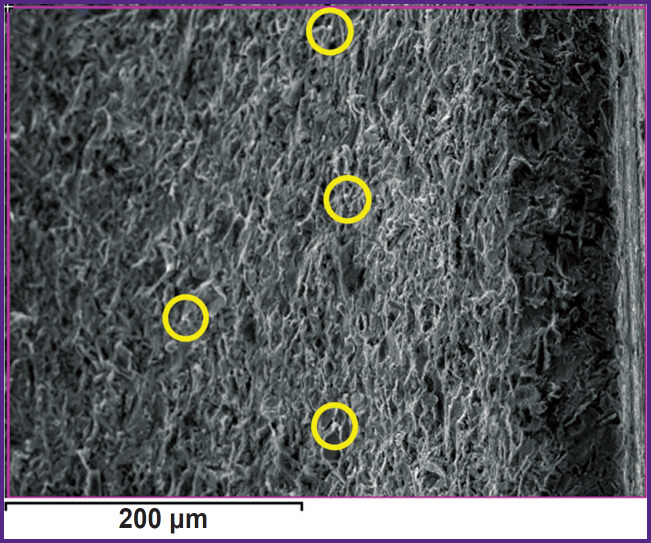
Image of the implant surface after sandblasting, with the marked corundum particles (yellow circles)

Examination of the implant surface after sandblasting using SEM showed a uniform distribution of corundum particles on the surface (marked with yellow circles in Figure 4).

The elemental composition of the surface layer (wt.%), determined for the analyzed areas similar to Figure 4, is provided in Table 1. According to the analysis, the main identified elements are Na, Al, P, Ca, and Ti. The average aluminum content determined using SEM images with dimensions of 1200×1200 and 400×400 μm is 2.67±0.79% (see Table 1).

**T a b l e 1:** Results of elemental analysis of the implant samples after sandblasting (wt.%)

SEM image	Na	Al	P	Ca	Ti
1	4.53	3.18	4.81	3.1	84.38
2	4.25	2.74	3.71	2.48	86.82
3	3.2	2.4	2.58	1.68	90.14
4	4.12	4.3	4.25	2.42	84.91
5	3.32	2.04	2.45	1.46	90.73
6	2.91	2.16	2.46	1.59	90.88
7	3.1	1.83	3.51	1.75	89.81
8	4.01	2.68	2.96	1.95	88.41
Mean value (total 100%)	3.69	2.67	3.34	2.05	88.25
Standard deviation	0.62	0.79	0.88	0.56	2.59

**Figure 5.**
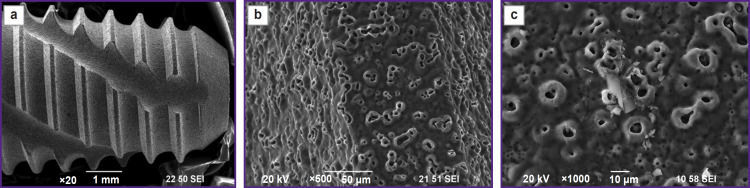
SEM image of the NCTi implant after sandblasting and subsequent PEO: (a) appearance; (b) implant surface; (c) corundum particle in the PEO coating

The appearance of the NCTi implant after the PEO is shown in Figure 5 (a). PEO creates an oxide layer 10–20 μm thick on the surface of a titanium implant [11]. The results of preliminary experiments to develop the method demonstrated that in order to obtain a uniform PEO coating on implants, surface pretreatment by sandblasting is required to create favorable conditions for microdischarge ignition. The formed coating consists of TiO_2_ anatase (60–70%) and TiO_2_ rutile (40–30%) [12].

Examination using SEM showed that the PEO coating on the implants had a rough surface with visible round pores (Figure 5 (b)). One should note that traces of mechanical sandblasting pretreatment were not visible, as the surface is oxidized, and the oxide is re-melted and re-solidified due to the impact of microdischarges during the PEO. The pores in the coating resulted from microdischarges; the diameter of large pores reached 5–6 μm.

**Figure 6.**
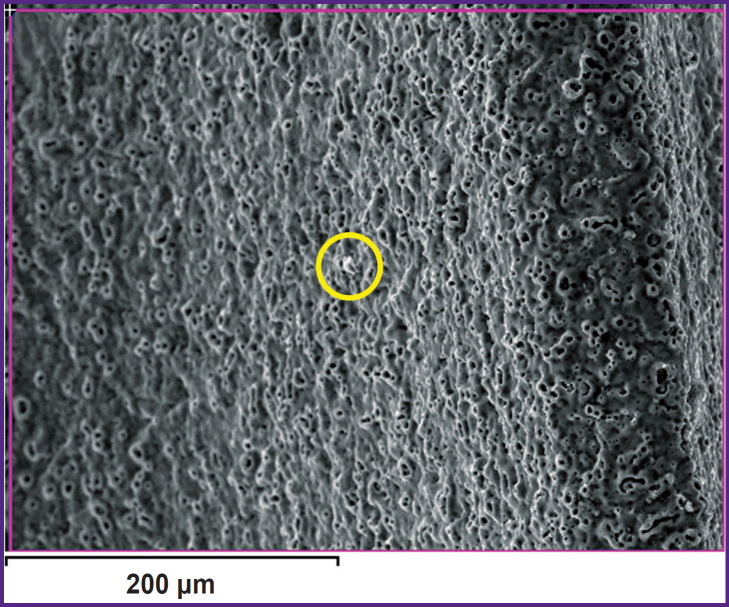
Image of the implant surface after sandblasting followed by PEO, with a marked corundum particle (a yellow circle)

Study of the coating on the implant surface by mapping the elemental composition after sandblasting followed by PEO confirmed the appearance of the corundum particles (Figure 6). When comparing Figures 4 and 6, one can note a decrease in the number of corundum particles in the microphotograph of the same size.

Table 2 shows the elemental composition of the surface layer (wt.%) determined from areas similar to those of Figure 6.

**T a b l e 2:** Results of elemental analysis of the implant samples after sandblasting and plasma electrolytic oxidation (wt.%)

SEM image	O	Na	Al	P	Ti
1	53.92	0.55	0.35	6.76	38.41
2	52.67	1.04	0.23	6.73	39.34
3	54.14	0.69	0.29	6.34	38.54
4	56.21	0.72	0.35	6.3	36.42
5	51.96	1.05	0.2	7.03	39.77
6	54.43	0.42	0.37	6.57	38.63
7	49.7	0.55	0.43	6.84	42.48
8	55.19	0.69	0.4	6.31	37.42
Mean value (total 100%)	53.53	0.71	0.33	6.61	38.82
Standard deviation	2.04	0.23	0.08	0.27	1.79

The main elements in the surface layer were O, Na, Al, P, and Ti. Apparently, the average aluminum content, determined from SEM images with the dimensions of 1200×1200 and 400×400 μm, was 0.33±0.08%.

**Figure 7.**
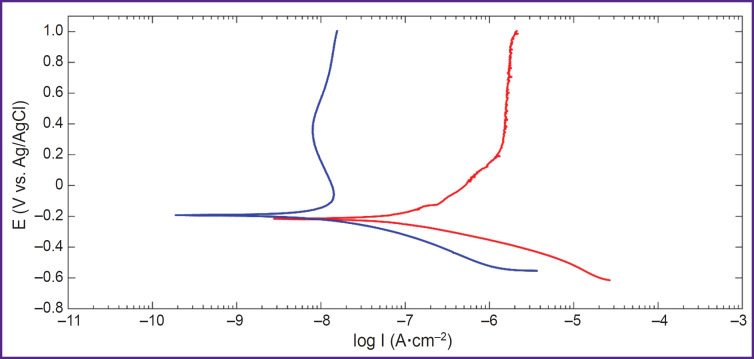
Polarization curves for samples after sandblasting (in red) and after sandblasting followed by PEO (in blue)

Figure 7 presents the results of the study of the electrochemical characteristics of the implants after sandblasting and sandblasting followed by PEO. The polarization curves of the samples (see Figure 7) demonstrated Tafel sections only on the cathode branches. A passivation section was seen on the anodic branch of the polarization curve of the PEO-coated sample, which follows from the decrease in current with the increasing potential ranging from –0.1 to 0.4 V.

The values of the calculated corrosion parameters are provided in Table 3. PEO leads to an upward shift in the value of the free corrosion potential E_corr_, thus passivating the surface. The PEO coating significantly improves the corrosion resistance due to an order of magnitude reduction in the corrosion current i_corr_. Polarization resistance is also increased, which is consistent with the values of the corrosion currents.

**T a b l e 3:** Results of the corrosion parameters calculation

Sample	E_corr_ (V)	i_corr_ (nA/cm^2^)	R_p_ (MΩ·cm^2^)
Sandblasting	–0.215±0.070	52.10±3.16	0.35±0.08
Sandblasting+PEO	–0.193±0.080	5.75±0.81	3.33±0.42

N o t e: data are provided as the mean values and standard deviation.

## Discussion

**Figure 8.**
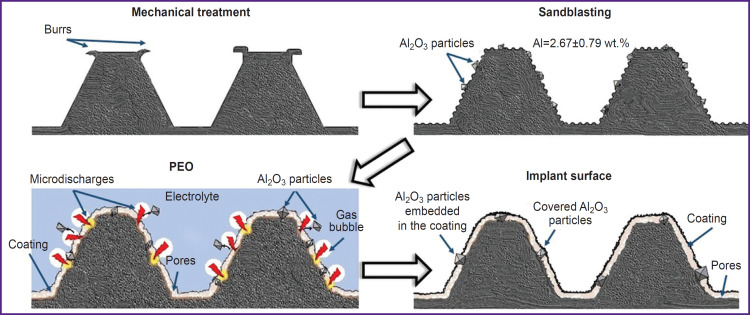
Schematic view of various implant surface treatment methods

The results of the study allow to propose a mechanism of impact of the considered methods of treating the implant surface on the appearance of the corundum particles. Figure 8 schematically shows various methods of the sequentially-used implant surface treatment: mechanical processing, sandblasting, and PEO.

Mechanical processing results in the implant shaping (Figure 8 shows a conventional thread profile of a dental implant), with inevitable burrs on the sharp edges of the thread. Sandblasting mechanically removes burrs, and a rough surface is formed. However, corundum particles get stuck in the surface layer. As a result, about 3 wt.% of aluminum is identified in the surface layer (Table 4).

**T a b l e 4:** Comparison of the aluminum content on the implant surface layer after various types of treatment (wt.%)

Method	Average Al content	Standard deviation
Sandblasting	2.67	0.79
Sandblasting and subsequent PEO	0.33	0.08

The subsequent PEO modifies the titanium metallic conductive surface through electrochemical anodic dissolution in combination with oxygen release during water electrolysis, which leads to formation of TiO_2_ oxides (rutile and anatase) on the surface [11]. This mechanism does not affect the Al_2_O_3_ corundum dielectric particles. However, the vapor-gaseous bubbles enclosing the plasma microdischarges show noticeable hydrodynamic impact on relatively large corundum particles in the surface layer. Such particles are mechanically released from the surface layer. However, some particles remain attached to the surface, and the PEO coating is formed around them. As a result, the aluminum content in the surface layer is reduced to 0.3 wt.% (see Table 4).

**Figure 9.**
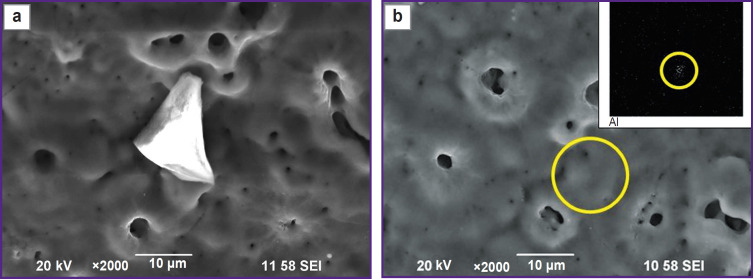
Image of a corundum particle: (a) particle emerging from the PEO coating; (b) particle covered by the PEO coating (a yellow circle); Al particle mapping in the upper right corner

This hypothesis is confirmed by the SEM results and elemental mapping. At magnification of 2000, corundum particles embedded in the surface layer can be seen in the PEO coating (Figure 9 (a)). Taking into account the fact that the corundum particle size is comparable to the thickness of the coating, some particles are not completely covered by the PEO coating (Figure 9 (a)), whereas some particles are covered (Figure 9 (b)). Thus, particles smaller than 10 μm are covered by the PEO coating, while larger particles are visible as inclusions in the PEO coating.

Finally, the PEO process significantly reduces the content of corundum particles in the surface layer after sandblasting; this allows to reduce the likelihood of the implant rejection, as such particles released from the surface of dental implants are involved in osseointegration, and have significant influence on this process. The emission of particles from the implant surface into the bone bed and their contact with tissue cells, as well as the immune system cells, result either in osseointegration with the bone structure formation, or in complications such as inflammation, allergic reaction, and idiosyncrasy [15].

## Conclusion

Sandblasting results in formation of a rough surface and mechanical removal of burrs seen after the shaping the implant by metal cutting. To obtain a uniform coating on all areas of the NCTi implant, surface pretreatment by sandblasting is required before plasma electrolytic oxidation.

Sandblasting with Al_2_O_3_ corundum particles 10– 30 μm in size leads to attachment of a small proportion of the particles to the implant surface layer. According to the results of elemental microanalysis, the weight fraction of aluminum in the implant surface layer is 2.67±0.79%. Plasma electrolytic oxidation facilitates reduction of the aluminum weight fraction on the implant surface layer to 0.33±0.08%. The PEO coating significantly improves corrosion resistance by an order of magnitude due to reduction in the corrosion current i_corr_.

The mechanisms to reduce the proportion of aluminum due to plasma electrolytic oxidation include mechanical removal of particles using microdischarges and masking of corundum particles in the thickness of the PEO coating.

Decrease in the proportion of aluminum introduced by corundum particles during sandblasting, as well as increase of the corrosion resistance of the surface layer by the plasma electrolytic oxidation method, have a positive effect on the titanium implant biocompatibility.
